# Supplementary material for: Resolving the full spectrum of human genome variation using Linked-Reads
Source: Genome Res. 2019 Apr;29(4):635–45. doi: 10.1101/gr.234443.118 (PMC6442396; doi:10.1101/gr.234443.118)
Supplement: Supplemental Material [file supp_gr.234443.118_Supplemental_longranger-2.2.zip › longranger-2.2.tar.bz2!/lr2.2-public/lib/stan/cmdstan/stan/doc/api/html/gamma_8hpp_source.html]

Stan: src/stan/prob/distributions/univariate/continuous/gamma.hpp Source File


|  |  |
| --- | --- |
|  | Stan  2.5.0  probability, sampling & optimization |


- Main Page
- Related Pages
- Namespaces
- Classes
- Files

- File List
- File Members

All Classes Namespaces Files Functions Variables Typedefs Enumerations Enumerator Friends Macros Pages

- src
- stan
- prob
- distributions
- univariate
- continuous

gamma.hpp

Go to the documentation of this file.

1 #ifndef STAN\_\_PROB\_\_DISTRIBUTIONS\_\_UNIVARIATE\_\_CONTINUOUS\_\_GAMMA\_HPP

2 #define STAN\_\_PROB\_\_DISTRIBUTIONS\_\_UNIVARIATE\_\_CONTINUOUS\_\_GAMMA\_HPP

3

4 #include <boost/random/gamma\_distribution.hpp>

5 #include <boost/random/variate\_generator.hpp>

6

7 #include <stan/agrad/partials\_vari.hpp>

8 #include <stan/math/error\_handling.hpp>

9 #include <stan/math/constants.hpp>

10 #include <stan/math/functions/multiply\_log.hpp>

11 #include <stan/math/functions/value\_of.hpp>

12 #include <stan/meta/traits.hpp>

13 #include <stan/prob/constants.hpp>

14 #include <stan/prob/traits.hpp>

15 #include <stan/prob/internal\_math.hpp>

16

17 namespace stan {

18

19  namespace prob {

20

43  template <bool propto,

44  typename T\_y, typename T\_shape, typename T\_inv\_scale>

45  typename return\_type<T\_y,T\_shape,T\_inv\_scale>::type

46  gamma\_log(const T\_y& y, const T\_shape& alpha, const T\_inv\_scale& beta) {

47  static const char\* function = "stan::prob::gamma\_log(%1%)";

48

49  using stan::is\_constant\_struct;

50  using stan::math::check\_not\_nan;

51  using stan::math::check\_positive\_finite;

52  using stan::math::check\_nonnegative;

53  using stan::math::check\_consistent\_sizes;

54  using stan::math::value\_of;

55

56  // check if any vectors are zero length

57  if (!(stan::length(y)

58  && stan::length(alpha)

59  && stan::length(beta)))

60  return 0.0;

61

62  // set up return value accumulator

63  double logp(0.0);

64

65  // validate args (here done over var, which should be OK)

66  check\_not\_nan(function, y, "Random variable", &logp);

67  check\_positive\_finite(function, alpha, "Shape parameter", &logp);

68  check\_positive\_finite(function, beta, "Inverse scale parameter", &logp);

69  check\_consistent\_sizes(function,

70  y,alpha,beta,

71  "Random variable","Shape parameter",

72  "Inverse scale parameter",

73  &logp);

74

75  // check if no variables are involved and prop-to

76  if (!include\_summand<propto,T\_y,T\_shape,T\_inv\_scale>::value)

77  return 0.0;

78

79  // set up template expressions wrapping scalars into vector views

80  VectorView<const T\_y> y\_vec(y);

81  VectorView<const T\_shape> alpha\_vec(alpha);

82  VectorView<const T\_inv\_scale> beta\_vec(beta);

83

84  for (size\_t n = 0; n < length(y); n++) {

85  const double y\_dbl = value\_of(y\_vec[n]);

86  if (y\_dbl < 0)

87  return LOG\_ZERO;

88  }

89

90  size\_t N = max\_size(y, alpha, beta);

91  agrad::OperandsAndPartials<T\_y, T\_shape, T\_inv\_scale> operands\_and\_partials(y, alpha, beta);

92

93  using boost::math::lgamma;

94  using stan::math::multiply\_log;

95  using boost::math::digamma;

96

97  DoubleVectorView<include\_summand<propto,T\_y,T\_shape>::value,is\_vector<T\_y>::value>

98  log\_y(length(y));

99  if (include\_summand<propto,T\_y,T\_shape>::value)

100  for(size\_t n = 0; n < length(y); n++) {

101  if (value\_of(y\_vec[n]) > 0)

102  log\_y[n] = log(value\_of(y\_vec[n]));

103  }

104

105  DoubleVectorView<include\_summand<propto,T\_shape>::value,is\_vector<T\_shape>::value>

106  lgamma\_alpha(length(alpha));

107  DoubleVectorView<!is\_constant\_struct<T\_shape>::value,is\_vector<T\_shape>::value>

108  digamma\_alpha(length(alpha));

109  for (size\_t n = 0; n < length(alpha); n++) {

110  if (include\_summand<propto,T\_shape>::value)

111  lgamma\_alpha[n] = lgamma(value\_of(alpha\_vec[n]));

112  if (!is\_constant\_struct<T\_shape>::value)

113  digamma\_alpha[n] = digamma(value\_of(alpha\_vec[n]));

114  }

115

116  DoubleVectorView<include\_summand<propto,T\_shape,T\_inv\_scale>::value,is\_vector<T\_inv\_scale>::value>

117  log\_beta(length(beta));

118  if (include\_summand<propto,T\_shape,T\_inv\_scale>::value)

119  for (size\_t n = 0; n < length(beta); n++)

120  log\_beta[n] = log(value\_of(beta\_vec[n]));

121

122  for (size\_t n = 0; n < N; n++) {

123  // pull out values of arguments

124  const double y\_dbl = value\_of(y\_vec[n]);

125  const double alpha\_dbl = value\_of(alpha\_vec[n]);

126  const double beta\_dbl = value\_of(beta\_vec[n]);

127

128  if (include\_summand<propto,T\_shape>::value)

129  logp -= lgamma\_alpha[n];

130  if (include\_summand<propto,T\_shape,T\_inv\_scale>::value)

131  logp += alpha\_dbl \* log\_beta[n];

132  if (include\_summand<propto,T\_y,T\_shape>::value)

133  logp += (alpha\_dbl-1.0) \* log\_y[n];

134  if (include\_summand<propto,T\_y,T\_inv\_scale>::value)

135  logp -= beta\_dbl \* y\_dbl;

136

137  // gradients

138  if (!is\_constant\_struct<T\_y>::value)

139  operands\_and\_partials.d\_x1[n] += (alpha\_dbl-1)/y\_dbl - beta\_dbl;

140  if (!is\_constant\_struct<T\_shape>::value)

141  operands\_and\_partials.d\_x2[n] += -digamma\_alpha[n] + log\_beta[n] + log\_y[n];

142  if (!is\_constant\_struct<T\_inv\_scale>::value)

143  operands\_and\_partials.d\_x3[n] += alpha\_dbl / beta\_dbl - y\_dbl;

144  }

145  return operands\_and\_partials.to\_var(logp);

146  }

147

148  template <typename T\_y, typename T\_shape, typename T\_inv\_scale>

149  inline

150  typename return\_type<T\_y,T\_shape,T\_inv\_scale>::type

151  gamma\_log(const T\_y& y, const T\_shape& alpha, const T\_inv\_scale& beta) {

152  return gamma\_log<false>(y,alpha,beta);

153  }

154

155

170  template <typename T\_y, typename T\_shape, typename T\_inv\_scale>

171  typename return\_type<T\_y,T\_shape,T\_inv\_scale>::type

172  gamma\_cdf(const T\_y& y, const T\_shape& alpha, const T\_inv\_scale& beta) {

173  // Size checks

174  if (!(stan::length(y) && stan::length(alpha) && stan::length(beta)))

175  return 1.0;

176

177  // Error checks

178  static const char\* function = "stan::prob::gamma\_cdf(%1%)";

179

180  using stan::math::check\_positive\_finite;

181  using stan::math::check\_not\_nan;

182  using stan::math::check\_consistent\_sizes;

183  using stan::math::check\_greater\_or\_equal;

184  using stan::math::check\_less\_or\_equal;

185  using stan::math::check\_nonnegative;

186  using stan::math::value\_of;

187  using boost::math::tools::promote\_args;

188

189  double P(1.0);

190

191  check\_positive\_finite(function, alpha, "Shape parameter", &P);

192  check\_positive\_finite(function, beta, "Scale parameter", &P);

193  check\_not\_nan(function, y, "Random variable", &P);

194  check\_nonnegative(function, y, "Random variable", &P);

195  check\_consistent\_sizes(function, y, alpha, beta,

196  "Random variable", "Shape parameter",

197  "Scale Parameter",

198  &P);

199

200  // Wrap arguments in vectors

201  VectorView<const T\_y> y\_vec(y);

202  VectorView<const T\_shape> alpha\_vec(alpha);

203  VectorView<const T\_inv\_scale> beta\_vec(beta);

204  size\_t N = max\_size(y, alpha, beta);

205

206  agrad::OperandsAndPartials<T\_y, T\_shape, T\_inv\_scale>

207  operands\_and\_partials(y, alpha, beta);

208

209  // Explicit return for extreme values

210  // The gradients are technically ill-defined, but treated as zero

211

212  for (size\_t i = 0; i < stan::length(y); i++) {

213  if (value\_of(y\_vec[i]) == 0)

214  return operands\_and\_partials.to\_var(0.0);

215  }

216

217  // Compute CDF and its gradients

218  using boost::math::gamma\_p\_derivative;

219  using boost::math::gamma\_p;

220  using boost::math::digamma;

221  using boost::math::tgamma;

222

223  // Cache a few expensive function calls if nu is a parameter

224  DoubleVectorView<!is\_constant\_struct<T\_shape>::value,

225  is\_vector<T\_shape>::value>

226  gamma\_vec(stan::length(alpha));

227  DoubleVectorView<!is\_constant\_struct<T\_shape>::value,

228  is\_vector<T\_shape>::value>

229  digamma\_vec(stan::length(alpha));

230

231  if (!is\_constant\_struct<T\_shape>::value) {

232  for (size\_t i = 0; i < stan::length(alpha); i++) {

233  const double alpha\_dbl = value\_of(alpha\_vec[i]);

234  gamma\_vec[i] = tgamma(alpha\_dbl);

235  digamma\_vec[i] = digamma(alpha\_dbl);

236  }

237  }

238

239  // Compute vectorized CDF and gradient

240  for (size\_t n = 0; n < N; n++) {

241  // Explicit results for extreme values

242  // The gradients are technically ill-defined, but treated as zero

243  if (value\_of(y\_vec[n]) == std::numeric\_limits<double>::infinity())

244  continue;

245

246  // Pull out values

247  const double y\_dbl = value\_of(y\_vec[n]);

248  const double alpha\_dbl = value\_of(alpha\_vec[n]);

249  const double beta\_dbl = value\_of(beta\_vec[n]);

250

251  // Compute

252  const double Pn = gamma\_p(alpha\_dbl, beta\_dbl \* y\_dbl);

253

254  P \*= Pn;

255

256  if (!is\_constant\_struct<T\_y>::value)

257  operands\_and\_partials.d\_x1[n]

258  += beta\_dbl \* gamma\_p\_derivative(alpha\_dbl, beta\_dbl \* y\_dbl)

259  / Pn;

260  if (!is\_constant\_struct<T\_shape>::value)

261  operands\_and\_partials.d\_x2[n]

262  -= stan::math::gradRegIncGamma(alpha\_dbl, beta\_dbl

263  \* y\_dbl, gamma\_vec[n],

264  digamma\_vec[n]) / Pn;

265  if (!is\_constant\_struct<T\_inv\_scale>::value)

266  operands\_and\_partials.d\_x3[n]

267  += y\_dbl \* gamma\_p\_derivative(alpha\_dbl, beta\_dbl \* y\_dbl) / Pn;

268  }

269

270  if (!is\_constant\_struct<T\_y>::value)

271  for (size\_t n = 0; n < stan::length(y); ++n)

272  operands\_and\_partials.d\_x1[n] \*= P;

273  if (!is\_constant\_struct<T\_shape>::value)

274  for (size\_t n = 0; n < stan::length(alpha); ++n)

275  operands\_and\_partials.d\_x2[n] \*= P;

276  if (!is\_constant\_struct<T\_inv\_scale>::value)

277  for (size\_t n = 0; n < stan::length(beta); ++n)

278  operands\_and\_partials.d\_x3[n] \*= P;

279

280  return operands\_and\_partials.to\_var(P);

281  }

282

283  template <typename T\_y, typename T\_shape, typename T\_inv\_scale>

284  typename return\_type<T\_y,T\_shape,T\_inv\_scale>::type

285  gamma\_cdf\_log(const T\_y& y, const T\_shape& alpha, const T\_inv\_scale& beta) {

286  // Size checks

287  if (!(stan::length(y) && stan::length(alpha) && stan::length(beta)))

288  return 0.0;

289

290  // Error checks

291  static const char\* function = "stan::prob::gamma\_cdf\_log(%1%)";

292

293  using stan::math::check\_positive\_finite;

294  using stan::math::check\_not\_nan;

295  using stan::math::check\_consistent\_sizes;

296  using stan::math::check\_greater\_or\_equal;

297  using stan::math::check\_less\_or\_equal;

298  using stan::math::check\_nonnegative;

299  using stan::math::value\_of;

300  using boost::math::tools::promote\_args;

301

302  double P(0.0);

303

304  check\_positive\_finite(function, alpha, "Shape parameter", &P);

305  check\_positive\_finite(function, beta, "Scale parameter", &P);

306  check\_not\_nan(function, y, "Random variable", &P);

307  check\_nonnegative(function, y, "Random variable", &P);

308  check\_consistent\_sizes(function, y, alpha, beta,

309  "Random variable", "Shape parameter",

310  "Scale Parameter",

311  &P);

312

313  // Wrap arguments in vectors

314  VectorView<const T\_y> y\_vec(y);

315  VectorView<const T\_shape> alpha\_vec(alpha);

316  VectorView<const T\_inv\_scale> beta\_vec(beta);

317  size\_t N = max\_size(y, alpha, beta);

318

319  agrad::OperandsAndPartials<T\_y, T\_shape, T\_inv\_scale>

320  operands\_and\_partials(y, alpha, beta);

321

322  // Explicit return for extreme values

323  // The gradients are technically ill-defined, but treated as zero

324

325  for (size\_t i = 0; i < stan::length(y); i++) {

326  if (value\_of(y\_vec[i]) == 0)

327  return operands\_and\_partials.to\_var(stan::math::negative\_infinity());

328  }

329

330  // Compute cdf\_log and its gradients

331  using boost::math::gamma\_p\_derivative;

332  using boost::math::gamma\_p;

333  using boost::math::digamma;

334  using boost::math::tgamma;

335

336  // Cache a few expensive function calls if nu is a parameter

337  DoubleVectorView<!is\_constant\_struct<T\_shape>::value,

338  is\_vector<T\_shape>::value>

339  gamma\_vec(stan::length(alpha));

340  DoubleVectorView<!is\_constant\_struct<T\_shape>::value,

341  is\_vector<T\_shape>::value>

342  digamma\_vec(stan::length(alpha));

343

344  if (!is\_constant\_struct<T\_shape>::value) {

345  for (size\_t i = 0; i < stan::length(alpha); i++) {

346  const double alpha\_dbl = value\_of(alpha\_vec[i]);

347  gamma\_vec[i] = tgamma(alpha\_dbl);

348  digamma\_vec[i] = digamma(alpha\_dbl);

349  }

350  }

351

352  // Compute vectorized cdf\_log and gradient

353  for (size\_t n = 0; n < N; n++) {

354  // Explicit results for extreme values

355  // The gradients are technically ill-defined, but treated as zero

356  if (value\_of(y\_vec[n]) == std::numeric\_limits<double>::infinity())

357  return operands\_and\_partials.to\_var(0.0);

358

359  // Pull out values

360  const double y\_dbl = value\_of(y\_vec[n]);

361  const double alpha\_dbl = value\_of(alpha\_vec[n]);

362  const double beta\_dbl = value\_of(beta\_vec[n]);

363

364  // Compute

365  const double Pn = gamma\_p(alpha\_dbl, beta\_dbl \* y\_dbl);

366

367  P += log(Pn);

368

369  if (!is\_constant\_struct<T\_y>::value)

370  operands\_and\_partials.d\_x1[n]

371  += beta\_dbl \* gamma\_p\_derivative(alpha\_dbl, beta\_dbl \* y\_dbl)

372  / Pn;

373  if (!is\_constant\_struct<T\_shape>::value)

374  operands\_and\_partials.d\_x2[n]

375  -= stan::math::gradRegIncGamma(alpha\_dbl, beta\_dbl

376  \* y\_dbl, gamma\_vec[n],

377  digamma\_vec[n]) / Pn;

378  if (!is\_constant\_struct<T\_inv\_scale>::value)

379  operands\_and\_partials.d\_x3[n]

380  += y\_dbl \* gamma\_p\_derivative(alpha\_dbl, beta\_dbl \* y\_dbl) / Pn;

381  }

382

383  return operands\_and\_partials.to\_var(P);

384  }

385

386  template <typename T\_y, typename T\_shape, typename T\_inv\_scale>

387  typename return\_type<T\_y,T\_shape,T\_inv\_scale>::type

388  gamma\_ccdf\_log(const T\_y& y, const T\_shape& alpha, const T\_inv\_scale& beta) {

389  // Size checks

390  if (!(stan::length(y) && stan::length(alpha) && stan::length(beta)))

391  return 0.0;

392

393  // Error checks

394  static const char\* function = "stan::prob::gamma\_ccdf\_log(%1%)";

395

396  using stan::math::check\_positive\_finite;

397  using stan::math::check\_not\_nan;

398  using stan::math::check\_consistent\_sizes;

399  using stan::math::check\_greater\_or\_equal;

400  using stan::math::check\_less\_or\_equal;

401  using stan::math::check\_nonnegative;

402  using stan::math::value\_of;

403  using boost::math::tools::promote\_args;

404

405  double P(0.0);

406

407  check\_positive\_finite(function, alpha, "Shape parameter", &P);

408  check\_positive\_finite(function, beta, "Scale parameter", &P);

409  check\_not\_nan(function, y, "Random variable", &P);

410  check\_nonnegative(function, y, "Random variable", &P);

411  check\_consistent\_sizes(function, y, alpha, beta,

412  "Random variable", "Shape parameter",

413  "Scale Parameter",

414  &P);

415

416  // Wrap arguments in vectors

417  VectorView<const T\_y> y\_vec(y);

418  VectorView<const T\_shape> alpha\_vec(alpha);

419  VectorView<const T\_inv\_scale> beta\_vec(beta);

420  size\_t N = max\_size(y, alpha, beta);

421

422  agrad::OperandsAndPartials<T\_y, T\_shape, T\_inv\_scale>

423  operands\_and\_partials(y, alpha, beta);

424

425  // Explicit return for extreme values

426  // The gradients are technically ill-defined, but treated as zero

427

428  for (size\_t i = 0; i < stan::length(y); i++) {

429  if (value\_of(y\_vec[i]) == 0)

430  return operands\_and\_partials.to\_var(0.0);

431  }

432

433  // Compute ccdf\_log and its gradients

434  using boost::math::gamma\_p\_derivative;

435  using boost::math::gamma\_p;

436  using boost::math::digamma;

437  using boost::math::tgamma;

438

439  // Cache a few expensive function calls if nu is a parameter

440  DoubleVectorView<!is\_constant\_struct<T\_shape>::value,

441  is\_vector<T\_shape>::value>

442  gamma\_vec(stan::length(alpha));

443  DoubleVectorView<!is\_constant\_struct<T\_shape>::value,

444  is\_vector<T\_shape>::value>

445  digamma\_vec(stan::length(alpha));

446

447  if (!is\_constant\_struct<T\_shape>::value) {

448  for (size\_t i = 0; i < stan::length(alpha); i++) {

449  const double alpha\_dbl = value\_of(alpha\_vec[i]);

450  gamma\_vec[i] = tgamma(alpha\_dbl);

451  digamma\_vec[i] = digamma(alpha\_dbl);

452  }

453  }

454

455  // Compute vectorized ccdf\_log and gradient

456  for (size\_t n = 0; n < N; n++) {

457  // Explicit results for extreme values

458  // The gradients are technically ill-defined, but treated as zero

459  if (value\_of(y\_vec[n]) == std::numeric\_limits<double>::infinity())

460  return operands\_and\_partials.to\_var(stan::math::negative\_infinity());

461

462  // Pull out values

463  const double y\_dbl = value\_of(y\_vec[n]);

464  const double alpha\_dbl = value\_of(alpha\_vec[n]);

465  const double beta\_dbl = value\_of(beta\_vec[n]);

466

467  // Compute

468  const double Pn = 1.0 - gamma\_p(alpha\_dbl, beta\_dbl \* y\_dbl);

469

470  P += log(Pn);

471

472  if (!is\_constant\_struct<T\_y>::value)

473  operands\_and\_partials.d\_x1[n]

474  -= beta\_dbl \* gamma\_p\_derivative(alpha\_dbl, beta\_dbl \* y\_dbl)

475  / Pn;

476  if (!is\_constant\_struct<T\_shape>::value)

477  operands\_and\_partials.d\_x2[n]

478  += stan::math::gradRegIncGamma(alpha\_dbl, beta\_dbl

479  \* y\_dbl, gamma\_vec[n],

480  digamma\_vec[n]) / Pn;

481  if (!is\_constant\_struct<T\_inv\_scale>::value)

482  operands\_and\_partials.d\_x3[n]

483  -= y\_dbl \* gamma\_p\_derivative(alpha\_dbl, beta\_dbl \* y\_dbl) / Pn;

484  }

485

486  return operands\_and\_partials.to\_var(P);

487  }

488

489  template <class RNG>

490  inline double

491  gamma\_rng(const double alpha,

492  const double beta,

493  RNG& rng) {

494  using boost::variate\_generator;

495  using boost::gamma\_distribution;

496

497  static const char\* function = "stan::prob::gamma\_rng(%1%)";

498

499  using stan::math::check\_positive\_finite;

500

501  check\_positive\_finite(function, alpha, "Shape parameter", (double\*)0);

502  check\_positive\_finite(function, beta, "Inverse scale parameter",

503  (double\*)0);

504

505  /\*

506  the boost gamma distribution is defined by

507  shape and scale, whereas the stan one is defined

508  by shape and rate

509  \*/

510  variate\_generator<RNG&, gamma\_distribution<> >

511  gamma\_rng(rng, gamma\_distribution<>(alpha, 1.0 / beta));

512  return gamma\_rng();

513  }

514

515  }

516 }

517

518 #endif

multiply\_log.hpp

traits.hpp

stan::agrad::tgamma

fvar< T > tgamma(const fvar< T > &x)

**Definition:** tgamma.hpp:15

stan::agrad::OperandsAndPartials::to\_var

T\_return\_type to\_var(double logp)

**Definition:** partials\_vari.hpp:138

stan::math::check\_positive\_finite

bool check\_positive\_finite(const char \*function, const T\_y &y, const char \*name, T\_result \*result)

**Definition:** check\_positive\_finite.hpp:12

error\_handling.hpp

stan::math::multiply\_log

boost::math::tools::promote\_args< T\_a, T\_b >::type multiply\_log(const T\_a a, const T\_b b)

Calculated the value of the first argument times log of the second argument while behaving properly w...

**Definition:** multiply\_log.hpp:51

stan::length

size\_t length(const T &)

**Definition:** traits.hpp:159

stan::DoubleVectorView

DoubleVectorView allocates double values to be used as intermediate values.

**Definition:** traits.hpp:358

stan::prob::gamma\_cdf\_log

return\_type< T\_y, T\_shape, T\_inv\_scale >::type gamma\_cdf\_log(const T\_y &y, const T\_shape &alpha, const T\_inv\_scale &beta)

**Definition:** gamma.hpp:285

partials\_vari.hpp

stan::agrad::value\_of

T value\_of(const fvar< T > &v)

Return the value of the specified variable.

**Definition:** value\_of.hpp:16

constants.hpp

stan::prob::gamma\_cdf

return\_type< T\_y, T\_shape, T\_inv\_scale >::type gamma\_cdf(const T\_y &y, const T\_shape &alpha, const T\_inv\_scale &beta)

The cumulative density function for a gamma distribution for y with the specified shape and inverse s...

**Definition:** gamma.hpp:172

stan::is\_vector

**Definition:** traits.hpp:83

stan::agrad::lgamma

fvar< T > lgamma(const fvar< T > &x)

**Definition:** lgamma.hpp:15

stan::agrad::OperandsAndPartials

A variable implementation that stores operands and derivatives with respect to the variable...

**Definition:** partials\_vari.hpp:76

traits.hpp

stan::return\_type::type

boost::math::tools::promote\_args< typename scalar\_type< T1 >::type, typename scalar\_type< T2 >::type, typename scalar\_type< T3 >::type, typename scalar\_type< T4 >::type, typename scalar\_type< T5 >::type, typename scalar\_type< T6 >::type >::type type

**Definition:** traits.hpp:406

stan::is\_constant\_struct

Metaprogram to determine if a type has a base scalar type that can be assigned to type double...

**Definition:** traits.hpp:57

stan::math::value\_of

double value\_of(const T x)

Return the value of the specified scalar argument converted to a double value.

**Definition:** value\_of.hpp:24

stan::prob::include\_summand

Template metaprogram to calculate whether a summand needs to be included in a proportional (log) prob...

**Definition:** traits.hpp:35

stan::agrad::OperandsAndPartials::d\_x2

VectorView< double \*, is\_vector< T2 >::value, is\_constant\_struct< T2 >::value > d\_x2

**Definition:** partials\_vari.hpp:83

stan::math::gradRegIncGamma

double gradRegIncGamma(double a, double z, double g, double dig, double precision=1e-6)

**Definition:** internal\_math.hpp:181

stan::math::check\_nonnegative

bool check\_nonnegative(const char \*function, const T\_y &y, const char \*name, T\_result \*result)

**Definition:** check\_nonnegative.hpp:52

stan::math::check\_consistent\_sizes

bool check\_consistent\_sizes(const char \*function, const T1 &x1, const T2 &x2, const char \*name1, const char \*name2, T\_result \*result)

**Definition:** check\_consistent\_sizes.hpp:13

stan::max\_size

size\_t max\_size(const T1 &x1, const T2 &x2)

**Definition:** traits.hpp:191

stan::agrad::gamma\_p

fvar< T > gamma\_p(const fvar< T > &x1, const fvar< T > &x2)

**Definition:** gamma\_p.hpp:16

internal\_math.hpp

stan::math::check\_less\_or\_equal

bool check\_less\_or\_equal(const char \*function, const T\_y &y, const T\_high &high, const char \*name, T\_result \*result)

**Definition:** check\_less\_or\_equal.hpp:52

stan::math::check\_not\_nan

bool check\_not\_nan(const char \*function, const T\_y &y, const char \*name, T\_result \*result)

Checks if the variable y is nan.

**Definition:** check\_not\_nan.hpp:56

stan::agrad::digamma

fvar< T > digamma(const fvar< T > &x)

**Definition:** digamma.hpp:16

stan::prob::gamma\_ccdf\_log

return\_type< T\_y, T\_shape, T\_inv\_scale >::type gamma\_ccdf\_log(const T\_y &y, const T\_shape &alpha, const T\_inv\_scale &beta)

**Definition:** gamma.hpp:388

value\_of.hpp

stan::prob::gamma\_log

return\_type< T\_y, T\_shape, T\_inv\_scale >::type gamma\_log(const T\_y &y, const T\_shape &alpha, const T\_inv\_scale &beta)

The log of a gamma density for y with the specified shape and inverse scale parameters.

**Definition:** gamma.hpp:46

stan::agrad::OperandsAndPartials::d\_x1

VectorView< double \*, is\_vector< T1 >::value, is\_constant\_struct< T1 >::value > d\_x1

**Definition:** partials\_vari.hpp:82

stan::agrad::OperandsAndPartials::d\_x3

VectorView< double \*, is\_vector< T3 >::value, is\_constant\_struct< T3 >::value > d\_x3

**Definition:** partials\_vari.hpp:84

constants.hpp

stan::agrad::log

fvar< T > log(const fvar< T > &x)

**Definition:** log.hpp:15

stan::VectorView

VectorView is a template metaprogram that takes its argument and allows it to be used like a vector...

**Definition:** traits.hpp:275

stan::prob::gamma\_rng

double gamma\_rng(const double alpha, const double beta, RNG &rng)

**Definition:** gamma.hpp:491

stan::math::check\_greater\_or\_equal

bool check\_greater\_or\_equal(const char \*function, const T\_y &y, const T\_low &low, const char \*name, T\_result \*result)

**Definition:** check\_greater\_or\_equal.hpp:58

stan::math::negative\_infinity

double negative\_infinity()

Return negative infinity.

**Definition:** constants.hpp:123


---

[ Stan Home Page ]

*© 2011–2014,
Stan Development Team.*
